# Prediction of major depressive episodes and suicide-related ideation over a 3-year interval among Japanese undergraduates

**DOI:** 10.1371/journal.pone.0201047

**Published:** 2018-07-19

**Authors:** Nobuyuki Mitsui, Satoshi Asakura, Keisuke Takanobu, Shinya Watanabe, Kuniyoshi Toyoshima, Yuki Kako, Yoichi M. Ito, Ichiro Kusumi

**Affiliations:** 1 Department of Psychiatry, Hokkaido University Graduate School of Medicine, Sapporo, Hokkaido, Japan; 2 Health Care Center of Hokkaido University, Sapporo, Hokkaido, Japan; 3 Department of Biostatistics, Hokkaido University Graduate School of Medicine, Sapporo, Hokkaido, Japan; Shinshu University School of Medicine, JAPAN

## Abstract

**Background:**

Suicide has been a leading cause of death among young adult populations in Japan. The aim of this study was to predict major depressive episodes (MDEs) and suicide-related ideation among university students using the Patient Health Questionnaire-9 (PHQ-9) and the Temperament and Character Inventory (TCI).

**Methods:**

The subjects were 2194 university students who completed the PHQ-9 and TCI in the 1st year (T1) and the PHQ-9 in the 4th year (T2) of university. Multiple logistic regression analysis was performed to predict MDEs and suicide-related ideation at T2. Moreover, recursive partitioning analyses were conducted to reveal the future risk of MDEs and suicide-related ideation.

**Results:**

The multiple logistic regression analyses of MDEs and suicide-related ideation at T2 revealed that depressive episodes, suicide-related ideation, and low self-directedness(SD) scores at T1 were significant predictors. The area under the curve of the model for MDEs was 0.858 and that for suicide-related ideation was 0.741. The recursive partitioning analyses revealed that a PHQ-9 summary score ≥15 at T1 predicted a high risk of MDEs at T2 and that both a PHQ-9 summary score ≥5 and a PHQ-9 #9 score ≥1 predicted a high risk of suicide-related ideation at T2.

**Conclusions:**

MDEs, suicide-related ideation, and low SD scores are significant predictors of future MDEs and suicide-related ideation.

## Introduction

For the past decade, suicide has been a leading cause of death among young adult populations in Japan. However, strategies for suicide prevention have not been sufficiently effective. A recent study of suicide completers among college students in Japan revealed that only 16.4% of suicide completers had been diagnosed previously with psychiatric disorders, and only 16.0% had received services through the university health centre [1]. Moreover, a recent systematic review reported that the prevalence of depression among university students was 30.6%, which was substantially higher than the prevalence in the general population [2]. As suicide prevention in the young adult population presents a host of challenges, studies on predictors of depressive episodes or suicide-related ideation are needed.

Personality traits are thought to be among the most important predictors of major depressive disorders and suicide-related behaviours. Previous prospective studies revealed that the neuroticism dimension of Eysenck’s theory of personality was a significant predictor of major depressive episodes (MDEs) [3, 4]. Concerning suicide-related behaviours and suicide-related ideation, personality traits including neuroticism, introversion and hopelessness were also reported as predictive factors [5]. Several recent studies used the Temperament and Character Inventory (TCI) in longitudinal designs for general populations with follow-up periods of one year or less [6–8], 4 years [9, 10], and 15 years [11]. These previous studies found that high harm avoidance and low self-directedness were significant predictive factors for future depressive symptoms.

The Patient Health Questionnaire-9 (PHQ-9) is a good screening tool for MDEs in various settings [12–14]. Unlike other self-rating assessment scales for depressive symptoms, the PHQ-9 evaluates the nine criteria for a major depressive episode in the Diagnostic and Statistical Manual of Mental Disorders, Fourth Edition (DSM-IV). In previous studies, this scale was also used for a longitudinal study [15] and to screen for major depression among college or university students [16–18].

Previously, we reported a cross-sectional study that used the TCI and the PHQ-9 among university student populations [19]. However, to the best of our knowledge, no study has tried to predict future depressive episodes and suicide-related ideation using the PHQ-9 in combination with the TCI.

This study aims to predict depressive episodes and suicide-related ideation among university students. In this study, we used the PHQ-9 to assess depressive episodes and the TCI to assess personality traits. We conducted a prospective observational study among university student cohorts.

## Materials and methods

### 1. Study sample

**Fig 1:**
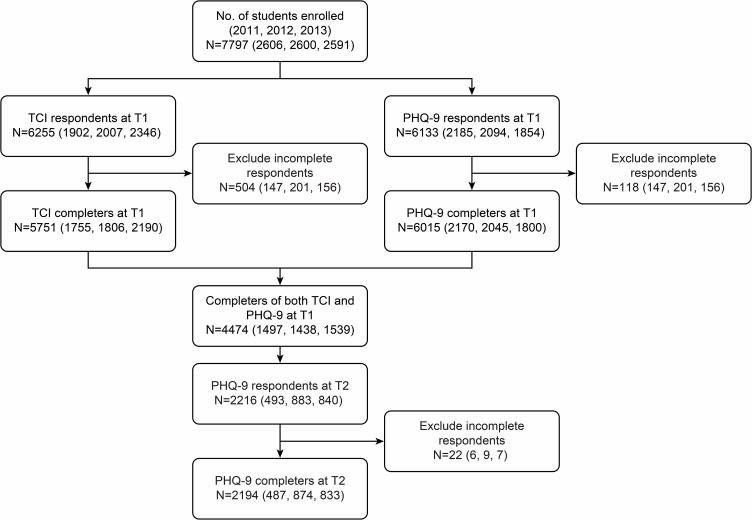
Participation flow. The number of participants in 2011, 2012, and 2013 are noted in brackets, following the total number of participants.

The PHQ-9 and the TCI were administered to university students who enrolled at Hokkaido University in April 2011, 2012, and 2013. The numbers of enrolled students in 2011, 2012, and 2013 were 2606, 2600, and 2591, respectively. After 3 years, the PHQ-9 was administered to the same students in April 2014, 2015, and 2016. We defined the first year as T1, and we defined the time of the retest 3 years later as T2. Among the students who enrolled in 2011, 2012, and 2013, the numbers of students who gave valid responses were 684 (26.2%), 1144 (44.0%), and 891 (34.4%), respectively. The definition of valid responses was completing all questions in the TCI and the PHQ-9 at T1 and T2. We excluded all students who gave incomplete responses to the TCI, the PHQ-9 at T1, or the PHQ-9 at T2 (Fig 1). Ultimately, 2194 (28.1%) students completed the TCI, the PHQ-9 at T1, and the PHQ-9 at T2. We defined these 2194 students as the subjects of our study. The prevalence of MDE at T1 and T2 were 2.0% and 2.3% respectively. These data were consistent with the data collected in 2010, which reported 2.9% prevalence [19]. These results indicated that a selection bias between T1 and T2 was low.

Written informed consent was obtained from all subjects prior to completion of the TCI, the PHQ-9 at T1, and the PHQ-9 at T2. This study was conducted in accordance with the ethical standards established in the 1964 Declaration of Helsinki (amended in Fortaleza, October 2013) and approved by the Ethical Committee of Hokkaido University Graduate School of Medicine.

### 2. Measurements

#### 2.1. Patient Health Questionnaire-9

The PHQ-9 is a self-report questionnaire of the Primary Care Evaluation of Mental Disorders (PRIME-MD). The validity of the PHQ-9 as a screening tool for major depressive episode has been confirmed in primary care settings. Two meta-analyses revealed the high sensitivity (0.80 and 0.77, respectively) and specificity (0.92 and 0.94, respectively) for the PHQ-9 [13, 20]. We used the Japanese version of the PHQ-9, which has high validity in primary care [21] and in psychiatric settings [14]. The PHQ-9 can diagnose the severity of depression by a summary score and depressive episodes by a diagnostic algorithm [12]. The diagnostic algorithm can distinguish between MDEs and other depressive episodes. PHQ-9 summary scores were divided into five categories: 0–4, 5–9, 10–14, 15–19, and 20 or greater [22]. Depressive symptoms were defined as a PHQ-9 summary score ≥5. When a subject responded of having “thoughts that you would be better off dead or of hurting yourself in some way” at least “several days” out of the week, he or she was regarded as having “suicide-related ideation”. The term “suicide-related ideation” encompasses ideas of both suicide and self-harm [23] and is coincident with a PHQ-9 #9 score ≥1.

#### 2.2. Temperament and character inventory

The TCI is a self-rating scale of personality based on the Psychological Model of Personality proposed by Cloninger [24]. The TCI consists of seven dimensions of personality, which are divided into four temperament and three character dimensions. The four temperament dimensions are novelty seeking (NS), harm avoidance (HA), reward dependence (RD), and persistence (P). The three character dimensions are self-directedness (SD), cooperativeness (C), and self-transcendence (ST). In this study, we used the 125-item Japanese version of the TCI with a 4-point scale. Kijima et al. reported that a 4-point scale was superior to a dichotomous scale in terms of internal consistency, as expressed by Cronbach’s α coefficient [25]. The Japanese version of the TCI is a valid and reliable measure of temperament and character for the university student population [26].

### 3. Statistical analyses

Demographic data were compared between female and male subjects by *t*-tests or chi-squared tests. ANOVA was applied to compare TCI scores among the non-depressive control (NC), other depressive episode (ODE), and MDE groups. Tukey’s honest significant difference (HSD) tests were used for post hoc analysis. We then performed multiple logistic regression analyses to create predictive models of future MDEs and suicide-related ideation. Five predictive variables, namely, gender, MDEs, suicide-related ideation, SD scores, and C scores, were used simultaneously. Moreover, recursive partitioning analysis, a type of decision tree analysis, was conducted to reveal criteria for future risk of MDEs and suicide-related ideation. Eight independent factors, namely, the PHQ-9 summary score at T1 and the NS, HA, RD, P, SD, C, and ST scores from the TCI, were used for future risk of major depression. Nine independent factors—the PHQ-9 summary score at T1, PHQ-9 #9 at T1; and the NS, HA, RD, P, SD, C, and ST scores from the TCI—were used to assess future risk of suicide-related ideation. In the first step, the subjects were divided into two groups according to whether their PHQ-9 summary scores were greater than 5. Then, the subjects were divided into four groups according to G^2^. The differences were considered significant at *P*<0.001 to compensate for the effects of large sample size and multiple comparisons. JMP Pro software version 13.0 was used for the analyses.

## Results

### 1. Demographic data and univariate analysis

The number of female subjects was 729 out of 2194 (36.1%). The mean age of the subjects at T1 was 19.2±0.9 (female, 19.2±0.8; male, 19.2±1.0) years old. The rate of subjects with MDEs at T1 was 2.0% (N = 44; female, 19; male, 25), and the mean PHQ-9 summary score was 3.5±3.4 (female, 3.7±3.4; male, 3.4±3.4), which was within the range that indicates a minimal level of depression severity [22]. The rate of subjects with suicide-related ideation at T1 was 5.1% (N = 110; female, 43; male, 67). Gender differences were not observed in mean age (*P* = 0.436), mean summary score on the PHQ-9 (*P* = 0.05), rate of MDE (*P* = 0.220), or rate of suicide-related ideation (*P* = 0.503) at T1.

Then, the PHQ-9 diagnostic algorithm was used to divide the subjects into three groups according to their scores at T2: NC, ODE, and MDE. Demographic data and comparisons of TCI scores between the three groups are shown in Table 1. The mean PHQ-9 summary score at T1 of the MDE group was significantly higher than that of the NC or the ODE group. The rate of subjects with suicide-related ideation in the MDE group was significantly higher than that in the ODE or the NC group.

**Table 1:** Comparisons of baseline data between NC, ODE, and MDE at T2.

	NC (1)	ODE (2)	MDE (3)	Statistics	P	(1) vs (2)	(1) vs (3)	(2) vs (3)
N	2058	85	51					
Gender*, f/m	731/1327	33/52	28/23	8.389	0.0151			
Age at T1^†^	19.2 (0.9)	19.2 (0.8)	19.3 (1.4)	0.293	0.7464			
*PHQ-9 at T1*								
Summary score^†^	3.2 (3.1)	6.0 (4.3)	8.9 (5.7)	102	<0.0001	<0.0001	<0.0001	<0.0001
NC*; N (%)	1963 (95.4%)	69 (81.2%)	31 (60.8%)	181.6	<0.0001			
ODE*; N (%)	71 (3.45%)	8 (9.4%)	8 (15.7%)					
MDE*; N (%)	24 (1.2%)	8 (9.4%)	12 (23.5%)					
SI*; N (%)	82 (4.0%)	7 (8.2%)	21 (41.2%)	146.5	<0.0001			
*TCI at T1*								
Novelty Seeking^†^	28.6 (6.4)	29.1 (7.7)	30.1 (7.2)	1.4	0.251	-	-	-
Harm Avoidance^†^	34.2 (9.1)	37.0 (10.1)	40.8 (10.0)	16.6	<0.0001	0.015	<0.0001	0.047
Reward Dependence^†^	26.3 (5.9)	24.4 (6.5)	24.8 (6.1)	5.6	0.004	0.01	0.186	0.911
Persistence^†^	8.3 (2.8)	7.9 (3.1)	8.0 (3.4)	0.8	0.434	-	-	-
Self-Directedness^†^	43.9 (9.1)	38.3 (9.0)	35.0 (8.7)	38.2	<0.0001	<0.0001	<0.0001	0.105
Cooperativeness^†^	48.6 (7.9)	46.3 (8.8)	44.9 (8.9)	8.2	0.0003	0.029	0.0037	0.589
Self-Transcendence^†^	14.1 (6.7)	15.3(7.3)	14.4 (8.1)	1.2	0.292	-	-	-
*PHQ-9 at T2*								
Summary score^†^	2.6 (2.7)	9.1 (2.4)	15.8 (3.0)	807.1	<0.0001	<0.0001	<0.0001	<0.0001
SI*; N (%)	71 (3.5%)	20 (23.5%)	38 (74.5%)	503.9	<0.0001			

Mean (Standard deviation) or number (percentage) are presented.

*Chi-square test

†ANOVA; Post-hoc test, Tukey’s honest significant difference test.

NC, Non-depressive controls; ODE, Other depressive episode; MDE, Major depressive episode

SI, Suicide-related ideation; TCI, Temperament and Character Inventory

PHQ-9, Patient Health Questionnaire-9

Regarding the TCI, the ANOVA revealed significant differences in HA, SD, and C. We then performed a post hoc analysis using the HSD test. No significant difference in TCI scores was found between the MDE group and the ODE group. The MDE group had significantly higher HA scores (*P*<0.0001), and lower SD scores (*P*<0.0001) than did the NC group. The ODE group had significantly lower SD scores (*P*<0.001) than did the NC group.

### 2. Multiple logistic regression analysis

To construct a predictive model of MDEs at T2, we used five factors obtained at baseline as independent factors for multiple logistic regression analysis (Table 2). MDEs and suicide-related ideation at T1 were the most significant predictors of MDEs at T2. The area under the curve (AUC) of this model was 0.858, and the adjusted R^2^ was 0.224. The sensitivity was 76.5%, and the specificity was 81.5%. Then, we constructed a predictive model of suicide-related ideation at T2 using the same five independent factors obtained at baseline. The AUC of this model was 0.741, and the adjusted R^2^ was 0.107. The sensitivity was 76.0%, and the specificity was 62.4%.

**Table 2:** Multiple logistic regression analysis for major depressive episodes and suicide-related ideation at T2.

Dependent factor	Independent factors at T1	β	SE	OR	*P* value	95% CI	
						Lower	Upper
Major depressive episode at T2	Gender	0.53	0.16	2.91	0.001	1.58	5.43
	Major or other depressive episodes	0.66	0.18	3.77	0.001	1.81	7.65
	Suicide-related ideation	0.89	0.18	5.90	<0.0001	2.84	11.93
	Self-directedness	-0.06	0.02	0.94	0.001	0.91	0.97
	Cooperativeness	-0.02	0.02	0.98	0.225	0.94	1.01
Suicide-related ideation at T2	Gender	0.26	0.10	1.68	0.009	1.14	2.47
	Major or other depressive episodes	0.30	0.14	1.81	0.045	1.01	3.13
	Suicide-related ideation	0.82	0.13	5.14	<0.0001	3.02	8.59
	Self-directedness	-0.04	0.01	0.96	0.001	0.94	0.98
	Cooperativeness	-0.03	0.01	0.97	0.016	0.95	0.99

### 3. Recursive partitioning analysis

**Fig 2:**
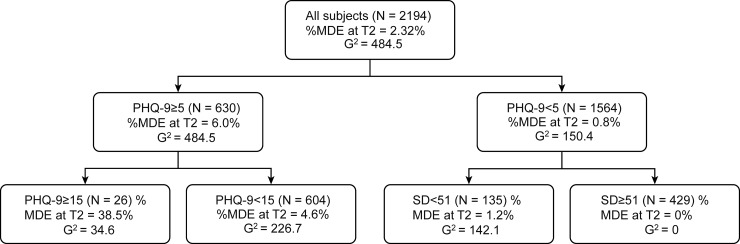
Recursive partitioning analysis for MDE at T2. Independent factors; PHQ-9 summary score at T1, NS, HA, RD, P, SD, C, and ST sores.

**Fig 3:**
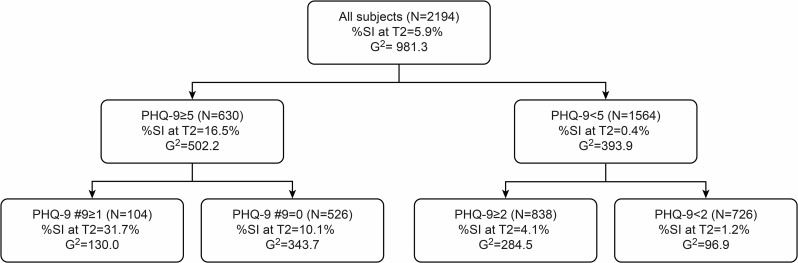
Recursive partitioning analysis for SI at T2. Independent factors; PHQ-9 summary score at T1, NS, HA, RD, P, SD, C, and ST sores. SI, suicide-related ideations.

To estimate future risk of MDE, we performed recursive partitioning analysis using the PHQ-9 summary score and all seven factors of the TCI as independent factors. At the first step, a PHQ-9 summary score ≤5 was selected for partitioning because a PHQ-9 summary score of 5 was the threshold of MDE at T2 (AUC = 0.801, sensitivity = 74.5%, specificity = 72.4%). A recursive partitioning analysis, as described in Fig 2, revealed that the subjects with the PHQ-9 summary scores ≥15 had a high rate of MDE at T2 (38.5%). On the other hand, the subjects with both a PHQ-9 summary score <5 and an SD score ≥51 had a low rate of MDE at T2 (N = 0). Concerning future risk of suicide-related ideation, the recursive partitioning analysis, as described in Fig 3, revealed that subjects with both a PHQ-9 summary score ≥5 and a PHQ-9 #9 score ≥1 had a high rate of PHQ-9 #9 scores ≥1 at T2 (31.7%). On the other hand, subjects with a PHQ-9 summary score <2 had a low rate of suicide-related ideation at T2 (N = 9, 1.2%).

## Discussion

The main finding of this study is that baseline MDEs and suicide-related ideation are the most significant predictors of future MDEs and suicide-related ideation. In regard to personality traits, low SD is another significant predictor of future depressive episodes and suicide-related ideation among newly enrolled students at the university. Conversely, high SD is a protective factor against future development of depressive episodes.

In the present study, MDEs at university enrolment (T1) were the most important predictive factor for depressive episodes 3 years later (T2). The prevalence of MDEs in this study was 2.0% at T1 and 2.3% at T2. However, 27.3% of the subjects with MDEs at T1 had MDEs at T2. The logistic regression analysis revealed that baseline MDEs were one of the most important predictors of future MDEs. A recent study of college students with depressive symptoms revealed that there was a persistent course of elevated depressive symptoms [27]. Moreover, a lifetime history of depression is one of the risk factors for future onset of MDEs among the general population [28]. Therefore, screening for MDEs using the PHQ-9 is meaningful not only at the time of testing but also in the future.

Regarding personality traits, low SD was the most significant predictor of both future depressive episodes and future suicide-related ideation in this study. Several previous studies have demonstrated that scores for the character dimension of SD are negatively associated with depressive symptoms [7, 29]. SD measures self-determination and the ability of an individual to control a situation in accordance with their individually chosen goals and values [24]. In a previous study, low SD scores were reported as a predictor of depressive symptoms after 12 months among Japanese college students [6]. Other previous longitudinal studies reported that high HA scores and low SD scores were substantial predictors of vulnerability to major depressive disorder. The previous longitudinal studies which were performed in the US or EU [7, 9, 10, 11] suggested that the association between low self-directedness and MDE or suicidal ideation were non-specific features for Japanese populations. In the present study, recursive partitioning analysis revealed that high SD scores were a substantial protective factor against future depressive episodes. This result was coincident with the results of a previous 12-month follow-up study, which reported that increased SD indicates an increase in coherence of personality that protects a person from depression [7].

Several limitations exist in this study. First, we used a self-reported questionnaire, the PHQ-9, to assess depressive episodes and suicide-related ideation. For diagnostic accuracy, we should perform a structured interview for each student to diagnose depressive episodes. However, it is difficult to apply these interviews to a large-scale screening test, such as the ones conducted in this longitudinal study. The second limitation of this study was that each subject was assessed at only two timepoints. We could not evaluate whether depressive symptoms persisted between those timepoints [27]. However, additional points of assessment would increase the dropout rate. Indeed, the present study, which assessed only two points, had an overall lower participation rate. Finally, in the present study, we could not distinguish between ideas of suicide and those of self-harm. We evaluated ideas of suicide or self-harm through item #9 of the PHQ-9. However, the relationship between suicide and self-harm behaviours is not clear in the college population. More than half of students who had engaged in self-harm behaviours reported never having considered or attempted suicide [30]. Therefore, ideas of self-harm are not always related to suicidal ideation.

## Conclusions

MDEs, suicide-related ideation, and low SD in the first year are significant predictors of future MDEs and suicide-related ideation among university students.
